# Sediment heterogeneity affects navigation by burrowers: a modelling study

**DOI:** 10.1098/rsif.2025.0113

**Published:** 2025-09-17

**Authors:** Xuejing Wang, Moey Rojas, Kelly Dorgan, Arghavan Louhghalam

**Affiliations:** ^1^ Department of Aerospace and Mechanical Engineering, The University of Arizona, Tucson, AZ, USA; ^2^ Department of Ecology and Evolutionary Biology, Cornell University, Ithaca, NY, USA; ^3^ The University of Texas at Austin Marine Science Institute, Port Aransas, TX, USA; ^4^ Department of Civil and Environmental Engineering, University of Massachusetts Lowell, Lowell, MA, USA

**Keywords:** burrowing, crack propagation, bioturbation, heterogeneous sediments, lattice element method

## Abstract

Worms extend burrows through muddy sediments by fracture, and the mechanics of crack propagation through heterogeneous sediments affects both navigation by burrowers and the release of particulate material, which is mixed through bioturbation. Crack propagation follows the path of least resistance or the lowest fracture toughness. Previous work showed that applying asymmetrical stress to burrow walls to simulate steering had minimal effect on crack propagation direction, suggesting that crack branching or the fusing of microcracks near the crack tip with the main burrow allows for burrowers to navigate by choosing between two directions. Here we use the lattice element method for modelling of fracture in heterogeneous materials to examine how fracture toughness, variability in fracture toughness and worm behaviours affect crack branching and microcracking. Experimental observations of worms burrowing in custom-built ant farm tanks support the modelling results that burrowing activities create microcracks both within the vicinity of the crack tip and in the surrounding sediment. In addition, hydraulic fracture driven by burrow irrigation reduces microcracking outside of the fracture process zone, potentially increasing the efficiency of burrowing. These results highlight the potential feedback between burrowing activities and sediment heterogeneity that characterize ecosystem engineering of sediment habitats by infaunal burrowers.

## 1. Introduction

Muddy marine sediments are elastic solids through which worms and other animals extend burrows by fracture [1]. Forces applied to the burrow walls are amplified at the tip of the burrow, and the elastic work done is stored in the material as elastic potential energy. When enough stress is focused at the crack tip that the elastic energy release rate reaches the fracture energy of the sediment, the burrow extends by fracture [2]. The mechanism of burrow extension by fracture, therefore, governs the locomotory performance of the burrower [3] and the energetic cost of burrowing [4]. This mechanism of burrow extension by fracture is limited to burrowers that are much larger than the grain size and to saturated, cohesive sediments that can be treated as a continuum [5,6].

Burrowing activities mix sediments and pore waters and modify the physical properties of sediments [7,8]. Developing a predictive understanding of this mixing, or bioturbation, has been limited by a lack of understanding of animal behaviours and how these behaviours affect sediments [9]. Recent work has introduced the idea that the mechanical properties of sediments are important in resisting bioturbation [10]. Considerable work has focused on how the community of infauna contribute to bioturbation [11], but recent work suggested that the fracture toughness of sediment is also potentially important in determining bioturbation rates in cohesive, muddy sediments [10]. We suggested that crack branching and microcracking around burrows that are extended through muddy sediments by fracture are important in releasing particles from the cohesive organic matrix to be mixed. Model results showed that fracture toughness is an important parameter in determining crack propagation direction and that variability in fracture toughness on small scales is important in enabling crack paths to deviate in directions of lower resistance.

The process of burrow extension by fracture is important in understanding how burrowing animals navigate as well. Surprisingly, previous modelling results suggested that steering a crack through a homogeneous material by applying asymmetric forces to the burrow walls is ineffective; instead, crack propagation follows the direction of lower fracture toughness [10]. These results raise questions about how worms navigate through their solid substrate; specifically, what strategies do burrowing animals use to extend burrows in a preferred direction and how do sediment properties affect the ease of navigation? We suggested that worms likely turn by creating crack branches and choosing between two directions [10]. Crack branching occurs when there are multiple directions a crack could propagate that have similar ratios of stress intensity factor, which drives fracture, to fracture toughness, the material resistance to fracture [10,12–14]. Here we build on our previous modelling work to explicitly test the effects of the material properties of sediments and of worm ‘behaviour’ on crack propagation direction and branching around a simulated worm extending a burrow by fracture.

In our previous work, we used the finite element analysis (FEA) software ABAQUS to show that fracture toughness affected the potential crack propagation direction, but we were unable to simulate iterative crack growth with this method [10]. FEA is widely used for modelling the mechanical response of materials to different loading conditions. However, modelling fracture with FEA is challenging, since accurately capturing the stress concentration at the tip of the crack requires high-resolution finite element meshes in the vicinity of the crack. Additionally, to correctly simulate crack propagation, the finite element mesh must be iteratively refined as the crack advances through the solid phase, introducing discontinuity. While advanced methods like the extended finite element method reduce the need for re-meshing around discontinuities, significant theoretical and numerical challenges remain in modelling dynamic fracture and fragmentation due to the inherently discrete nature of these processes [15–17]. To address these challenges, we adopt a discrete modelling approach. The discrete element method (DEM) is effective for modelling dry, granular materials [18]; however, it is less suited for saturated, cohesive sediments like mud. We, therefore, use the lattice element method (LEM) [19–22] to model fracture in these materials where cohesion dominates and particle-scale interactions are less critical. Our simulation platform relies on a potential of mean force (PMF) approach to LEM [23,24] and incorporates a newly developed hybrid energy-based method for simulating fracture [25]. This approach has shown great potential for modelling crack propagation and initiation of microcracks in heterogeneous materials [25,26].

Microcracking is the initiation and growth of new cracks in regions around the crack tip in which the stored elastic energy is higher than the fracture toughness [27]. Resistance to crack initiation, the first step in microcracking, is lower in heterogeneous than in homogeneous materials; cracks initiate in very small regions with lower fracture toughness [28]. In the context of burrowing, microcracking can be advantageous if the microcracks occur in front of the crack tip and eventually fuse with the main crack. These microcracks can extend the main crack or lead to crack branching, providing the worm with options for navigation, and can potentially facilitate the formation of a long-term cylindrical burrow. If microcracks occur outside of the zone in front of the burrow, they are unlikely to benefit the burrower in navigation; rather, the release of stored energy during crack formation likely decreases the energetic efficiency of burrowing. Microcracking outside of the zone in front of the burrow would contribute to sediment heterogeneity; initiation of a crack in a low-toughness region effectively reduces the toughness to zero. Extensive microcracking could also release particles to be mixed through bioturbation.

In this study, we use a hybrid energy-based approach [25] to simulate iterative crack propagation, including microcracking and crack branching, in randomly generated heterogeneous sediments. We generated realizations of sediments with different coefficients of variation (CoV) of fracture toughness and different mean fracture toughness (KIc), where the overbar denotes the mean value of the random field, to test the hypotheses that (H1) more variability in fracture toughness will result in more crack branching and microcracking, and (H2) when more energy is available for fracture, there will be more crack branching and microcracking. Our previous modelling showed that fracture toughness was much more important than stiffness in affecting crack propagation direction [10]; in these simulations, the elastic modulus (*E*) is kept constant, and the fracture energy, the critical energy release rate, is directly proportional to $K_{Ic}^{2}$ [27]. Infaunal animals use undulation and peristalsis to pump oxygenated surface waters down into their burrows [29], and it is likely that these movements also drive fluid flow towards the burrow tip during burrow formation. We also applied different pressure patterns to the crack walls to examine the hypothesis (H3a): that irrigation behaviours applying more stress at the crack tip, resulting in hydraulic fracture, will focus crack propagation around the crack tip and decrease microcracking in regions away from the crack tip. While previous studies have used DEM to directly model hydraulic fracture [18], our focus here is on the fracture process in low-permeability sediments, so we simulate only the expected change in stress distribution around the crack tip. Although our previous work showed that applying asymmetric stress to the burrow walls did not substantially change the direction of crack propagation in a homogeneous material, here we test the hypothesis (H3b): that interactions between asymmetric stress and heterogeneity in fracture toughness may steer the crack path over multiple crack propagation events. We compare our modelling results to observations of worms burrowing in natural sediments, using a novel ant farm tank to enable observations of fracture patterns while the worm burrows. Understanding these interactions between sediment properties, specifically the variability in fracture toughness, and burrower behaviour has important implications for understanding both navigation in sediments and the mechanisms by which animals modify sediment structure.

## 2. Methods

### 2.1. Lattice element method simulations of crack propagation in heterogeneous sediments

**Figure 1.**
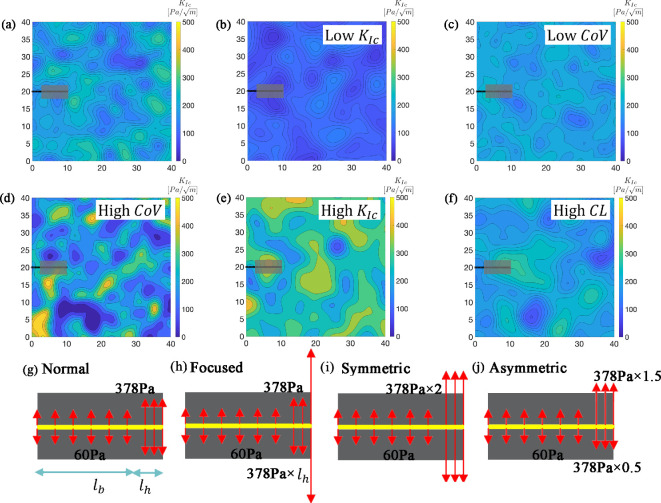
Top two rows: realizations of fracture toughness field for (a) reference sediment (KIc=200 $\text{Pa}/\sqrt{\text{m}}$, CoV = 0.2, CL = 0.05 m); (b) sediment with lower toughness (KIc=100 $\text{Pa}/\sqrt{\text{m}}$, CoV = 0.2, CL = 0.05 m); (c) sediment with low variability of fracture toughness (KIc=200 $\text{Pa}/\sqrt{\text{m}}$, CoV =0.1, CL = 0.05 m); (d) sediment with high variability of fracture toughness (CoV =0.4); (e) sediment with higher toughness (KIc=300 $\text{Pa}/\sqrt{\text{m}}$); and (f) sediments with higher CL (CL =0.10 m). Bottom row: different patterns of pressure applied to crack walls by simulated worms. A uniform pressure of 60 Pa is applied by the body ($l_{b}$ = 100 mm) in all loading cases, while the pressure applied by the worm’s head ($l_{h}$ = 12.5 mm) varies. (g) In the ‘Normal’ case, a symmetric uniform pressure of 378 Pa is applied. Modifications include (h) the impact of an additional focused force at the tip, simulating hydraulic pressure as the worm moves forward, driving fluid towards the tip of the crack; (i) a higher stress (equal to the integral of pressure in (h)) is applied along $l_{h}$; and (j) an additional force applied to one burrow wall, simulating the worm pushing harder on one side to steer the crack.

To test these hypotheses, we perform numerical simulations using the PMF-based LEM to model the crack propagation in heterogeneous sediments by burrowing worms. The reader is referred to [25] and electronic supplementary material, appendix S1, for a comprehensive explanation of the approach’s underlying methodology. We simulate a two-dimensional (2D) heterogeneous sediment with an initial worm-sized crack on one side of the sediment subjected to pressure from a worm’s dorsal and ventral surfaces (figure 1g–j), following experimental measurements on the annelid, *Allita virens*, burrowing in gelatin, an analogue for muddy sediments [2] and our previous modelling studies [3,10]. These worms are 8−10 cm long, and 5−6 mm thick [2] and simulated with an initial crack of length 11.25 cm, slightly exceeding worm length. The simulations are performed on samples of size 40 cm by 40 cm (figure 1), 4× worm length and sufficiently large to statistically represent sediment heterogeneity. We generate 160 independent realizations of 2D fracture toughness fields following a Weibull distribution with mean fracture toughness of 100, 200 and 300 ($\text{Pa}/\sqrt{\text{m}}$), following the ranges of fracture toughness values measured in sediments from Mobile Bay, AL [30]. CoV and correlation lengths (CLs) of fracture toughness have not been quantified from natural sediments, but we followed our previous modelling study and used CoV varying between 0.1 and 0.4 (dimensionless) and two CLs of 0.05 and 0.1 m (see electronic supplementary material, appendix S2 table S2, for detailed information on the number of realizations for each simulation and figure 1). The effect of mesh resolution on stress distribution was evaluated through a series of LEM simulations with varying lattice sizes to ensure mesh-independent results. The domain is discretized using a square lattice of unit size 0.25 cm, as further refinement did not significantly improve accuracy. This resolution is also appropriate for capturing material heterogeneity, given the CLs used in this study. In our simulations, we assume isotropy and a 2D plain strain condition [3], thereby neglecting three-dimensional stress effects. We also assume a quasi-static loading condition, which neglects dynamic effects.

The worm, illustrated by a black line in figure 1a–f, applies pressure to the crack surface, according to previous measurements of forces using photoelastic stress analysis [2] (‘Normal’), and modified to characterize the impacts of worm ‘behaviours’. These modifications include (i) assuming that worms also drive fluid forward in their burrow to apply a more focused stress at the tip (‘Focused’), (ii) that worms apply more force to one side of the burrow than the other in an attempt to steer (‘Asymmetric’), and, to allow more direct comparison, (iii) apply equivalent integrated stress to the focused and asymmetric cases but symmetrically along the ‘head’ region of the crack (‘Symmetric’; figure 1g–j). Our simulations do not explicitly model pore pressure, fluid flow or hydro-mechanical coupling. Instead, to maintain computational efficiency, the mechanical effects of worm-generated fluid pressure are approximated using equivalent nodal forces applied at the crack edges and tip. This pressure is applied incrementally to extend the crack quasi-statically. A free boundary condition is applied at the edges of the domain, and both elastic modulus, *E*, and Poisson’s ratio, ν, are constant. The hybrid energy-based LEM framework [25] directly applies the Griffith criterion of linear elastic fracture mechanics (LEFM): a crack propagates when the energy release rate (G, energy released per unit area (J m^−2^)) exceeds the fracture energy $G_{c}$, which is intrinsic to a material. Given the constant elastic modulus, the spatial variation in fracture energy is translated into variability in fracture toughness $K_{Ic}$ via Irwin’s relation [31], i.e. $K_{Ic}=\sqrt{EG_{c}/(1-\nu^{2})}$. Once the worm applies enough pressure to initiate fracture, the fracture propagates, releasing energy until the energy available for fracture (i.e. energy release rate G) is lower than the sediment fracture energy $G_{c}$ and the fracture stops. At each loading increment, the energy driving the fracture is compared to the fracture toughness, and bonds attached to the particles with driving force exceeding fracture toughness are removed, and the burrow extends forward. This process is repeated until equilibrium is achieved. The pressure is then increased in the next loading increment. In the process, if a crack branched at the tip, the branching event is logged, and a direction is randomly selected as the main crack path.

**Figure 2.**
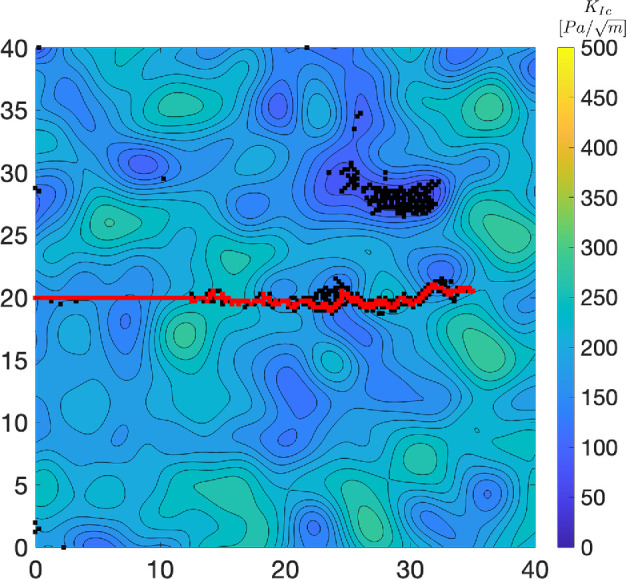
Crack patterns and burrow extension through the 40 cm × 40 cm sediment model with heterogeneous fracture toughness ( KIc= 200 $\text{P}\text{a}\;/\sqrt{\text{m}}$, CoV = 0.1, CL = 0.05 m). The crack extends from left to right by incrementally applying pressure (‘Normal’ case) along 100 mm from the tip of the crack. In addition to microcracks (black) in the vicinity of the main crack path (red), microcracks occur away from the major crack path in regions of lower fracture toughness.

Once the pressure application and resulting fracture reach equilibrium, the burrowing cycle is complete, and the worm moves forward into the newly created void and applies pressure to the new burrow walls [2,6]. Throughout the progression of the fracture, the major crack is defined as that formed in front of the worm and that forms the path of movement (red regions in figure 2). Microcracks are characterized as scattered cracks that are either not contiguous to the major crack or that are not followed by the simulated worm (black regions in figure 2).

### 2.2. Experimental observations of fracture around burrowing worms

To evaluate whether burrow extension by worms burrowing in natural sediments is consistent with our model predictions, we analysed videos of the annelid, *Alitta virens,* burrowing in muddy sediments in a ‘Laminar Ant Farm’ tank. These custom-built tanks suspend a 1.3-cm-thick vertical layer of mud (2× the diameter of the worms used) between two 1.3-cm-thick layers of transparent gelatin that mimics some of the elastic properties of muddy sediments [2], reduces wall effects [5] and allows for concurrent visualization of both a burrowing worm and the surrounding sediment at depth. The middle layer of the tanks was filled with homogenized muddy sediment from either Point aux Pins, AL (30.384 N, −88.312 W) or Mobile Bay, AL (30.25241, −88.011) that was allowed to settle for at least 72 h. Worms were purchased from the Phil Harrington Bait Company (Woolwich, ME), sent to the Dauphin Island Sea Lab via overnight FedEx and kept in muddy sediment collected from Point aux Pins until the time of video recording. *Alitta virens* burrows via eversion and retraction of its pharynx [2]. We recorded videos of 38 individual worms with a Nikon D5300 or Z6 DSLR camera with a Nikkor 18−140 1:3.5−5.6 or Nikkor 24−70/4 S lens, respectively. We then identified segments in which worm behaviour and the crack-shaped burrow were visible. Evidence of crack branching and microcracking around the crack tip has been previously described [10], so here we searched for evidence of (i) microcracking or crack propagation occurring away from the main burrow crack but presumably due to the burrowing worm and (ii) burrow extension consistent with ‘hydraulic fracture’, or crack propagation driven by increased water pressure in front of an everted pharynx at the crack tip (consistent with figure 1h). For (i), we looked for examples in which we could view both the position of the entire main burrow crack and see crack propagation separate from the main burrow crack occurring synchronously with pharynx eversions. For (ii), we looked for clear evidence of crack propagation occurring far in front of the worm’s pharynx, as well as evidence that water movement towards the crack tip was occurring concurrently with crack propagation.

## 3. Results

### 3.1. More microcracking occurs in materials with higher variability in fracture toughness

Our simulations of crack propagation and burrow extension through heterogeneous sediment show that branching and microcracking are not only located close to the burrow but also appear in regions with low fracture toughness that are far away from the anterior end of the worm (figure 2).

**Figure 3.**
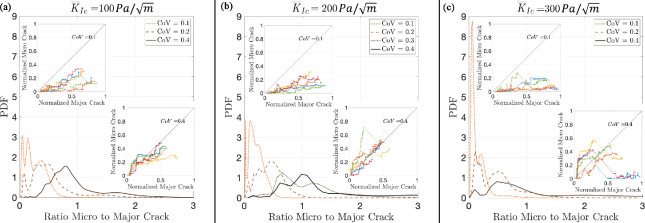
PDF of the ratio of fracture surface corresponding to microcracks to that of the major crack for different fracture toughness mean values (KIc) and CoV. The insets show the instantaneous crack length of microcracks versus the major crack for five different realizations (different colours) as the crack advances through the heterogeneous sediment, both normalized by the final total crack length. The curves above the y=x line indicate the dominance of microcracks, whereas curves below the y=x line indicate the dominance of the major crack.

To test hypothesis H1—more variability in fracture toughness results in more microcracks—we simulated burrow extension by crack propagation in sediments with different CoV of fracture toughness. We determined the crack length for major and microcracks over the duration of the simulation and presented the ratio of microcrack-to-major crack length to standardize the relative importance of microcracks in each simulation. The probability density functions (PDFs) of the ratio of microcrack-to-major crack length show relatively fewer microcracks when CoV is low across all mean values of fracture toughness (figure 3). Simulations with higher CoV show both more microcracks on average, consistent with our hypothesis, and a longer tail for the distribution, indicating that in some cases the microcrack length is several times that of the major crack (figure 3). This result is consistent irrespective of the mean value of $K_{Ic}$. Each PDF is obtained using the simulation results across 10 different realizations of each fracture toughness field (see electronic supplementary material, appendix S3 figures S2-S9).

This simulation also tests hypothesis H2: that more driving force leads to more branching of the cracks and microcracks. According to LEFM, crack propagation occurs when the stress intensity or energy release rate (driving force), respectively, reaches the fracture toughness ($K_{Ic}$) or fracture energy ($G_{c}$) of the material (both are material properties and are in essence the force resisting fracture). Fracture therefore depends on the ratio of driving force to the resisting force and not to their absolute values. Here, we vary the resisting force ($K_{Ic}$) while keeping the driving force (applied by the simulated worm) constant, so lower mean ($K_{Ic}$) corresponds to more energy available for fracture. Our results indicate that varying $K_{Ic}$, and therefore the relative amount of energy available for fracture, has surprisingly minimal impact on crack patterns (figure 3).

### 3.2. Crack branching frequency peaks at intermediate gradient in fracture toughness

**Figure 4.**
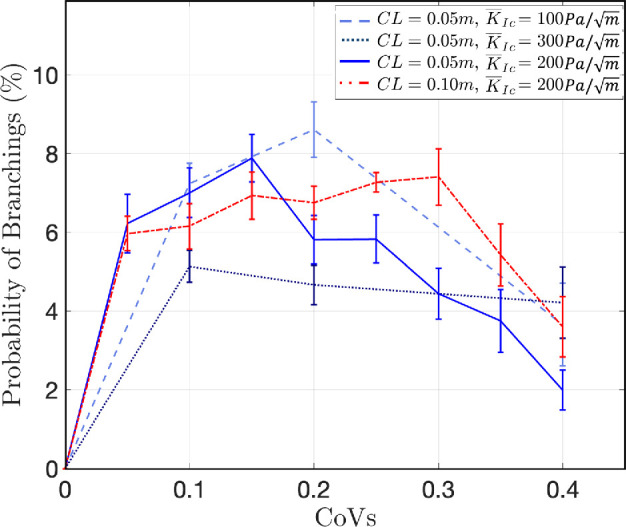
Probability of crack branching as a function of the CoV of fracture toughness plotted for samples with different mean value of toughness $K_{Ic}$ and CL. The error bars represent the standard error calculated across 10 realizations.

We hypothesized that the mean and variability of fracture toughness would affect both microcracking and crack branching. At each instance in the crack propagation simulations in which the energy release rate (driving force) in two or more directions (almost) simultaneously approaches the material fracture energy (resisting force), we record the possible occurrence of crack branching. One direction is then randomly selected from those with maximum energy release rate to be the main crack, and the other directions are denoted as microcracks. The probability of branching is calculated as the number of crack branching occurrences divided by the total number of crack propagation increments (figure 4). As observed in figure 4, the probability of crack branching peaks at intermediate CoV values in all of our simulated materials. Note that in homogeneous materials (CoV = 0), the energy release rate is always maximum along the crack direction, causing the crack to propagate straight in the direction of least resistance without any branching (the origin in figure 4). As the heterogeneity of sediments increases, crack propagation events in which energy release rates in multiple directions are equal or very close in magnitude occur more frequently, thereby increasing the likelihood of crack branching. When the sediment heterogeneity increases further, the probability of branching decreases, potentially because the direction of least resistance can become more pronounced. Interestingly, the CoV at which crack branching peaks does not appear to be consistent across different mean values of fracture toughness; rather, materials with lower KIc appear to have a more pronounced peak in branching frequency at a higher CoV value among samples with the same CL. The shift of the peak towards lower CoV for higher KIc should be interpreted with caution, as the same CoV around a higher mean value will have greater local variability. Crack propagation direction depends on variability in the immediate vicinity of the crack tip; thus, it follows that crack branching would depend on the local gradient in fracture toughness rather than the variability across the entire simulated region.

To better understand the peak in crack branching with increasing CoV of fracture toughness, we varied the fracture toughness CL, a measure of the distance over which the fracture toughness field is correlated, and assessed the impacts on the probability of branching. A longer CL results in a less steep gradient in fracture toughness for materials with the same CoV, therefore altering the local variability of fracture toughness but not the global variability. Increasing CL shifts the peak of branching probability to higher CoVs (figure 4). Similarly, greater global heterogeneity (i.e. higher CoV) is needed to produce the same level of local variability that promotes branching.

### 3.3. Focusing stress at the crack tip reduces microcracking

**Figure 5.**
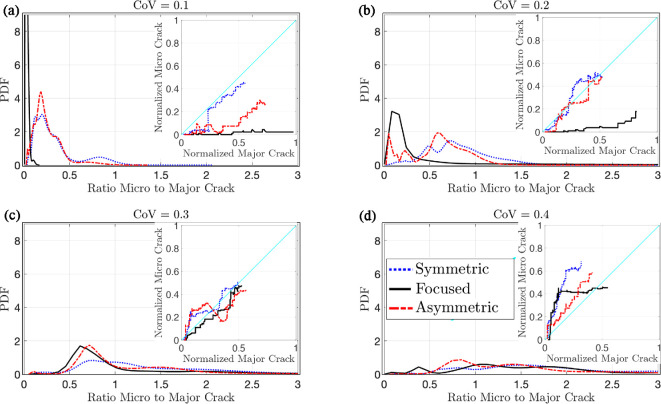
The PDF of the ratio of microcrack-to-major crack lengths subjected to three load patterns at four CoVs of toughness. Analogous to figure 3, each PDF is derived from five realizations of KIc=200 Pa$/\sqrt{\text{m}}$ and incorporates all crack propagation instances as the burrow extends through the sediment. The inset depicts the instantaneous plot of microcrack length versus the major crack length normalized by the total fracture surface for one of the five realizations subjected to three different loading conditions as the crack advances (other realizations are shown in electronic supplementary material, appendix S6, figures S12–S15).

To test hypothesis H3a—that concentration of pressure at the crack tip, e.g. through hydraulic pressure between the anterior of the worm at the crack tip, will reduce microcracking—we conduct fracture simulations with different load conditions applied to the burrow walls on samples with a mean $K_{Ic}=200$ Pa$/\sqrt{\text{m}}$ (figure 1h–j). The PDF of the ratio of microcrack-to-major crack length shows a sharp peak near 0 for the case of focused stress at the crack tip at a CoV of 0.1, indicating very minimal microcracking, in contrast to higher ratios for less focused stresses (figure 5a). This effect becomes less pronounced at higher CoVs of fracture toughness, with high occurrence of microcracking across all loading conditions at CoV of 0.4 (figure 5). It is also observed that concentrating the pressure at the tip has led to more instances of merging of microcracks into the major crack (electronic supplementary material, appendix S6). Furthermore, sharp increases occur when the major crack tip reaches a region of high $K_{Ic}$.

**Figure 6.**
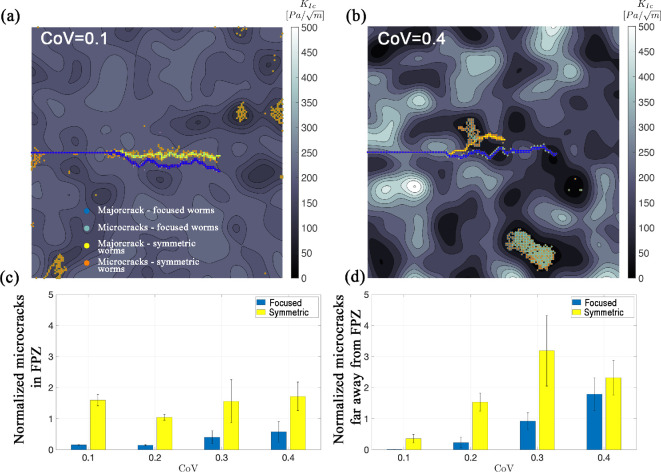
Crack patterns corresponding to two load conditions (i.e. symmetric and focused) shown for two different simulated sediment materials: (a) KIc=200 Pa$/\sqrt{\text{m}}$, CoV =0.1; (b) KIc=200 Pa$/\sqrt{\text{m}}$, CoV =0.4. In the CoV=0.1 sample, both simulations stop when the worm burrows through three-fourths of the sample length. In the CoV = 0.4 sample, the crack induced by the focused worm extends beyond three-fourths of the sample length; for direct comparison, however, the results up to three-fourths of the sample length are used for statistical analysis. The bar charts in (c,d) show the surfaces corresponding to microcracks within and away from the FPZ, respectively, both normalized by the surface of the major crack; the error bars represent the standard error obtained from five samples.

The prevalence of microcracks when subjected to symmetric loading compared to the focused loading is shown for two realizations with low and high CoVs in figure 6a,b, respectively (see electronic supplementary material, appendix S6, videos S1–S4, for additional realizations). The decline in microcracking under focused loads appears to occur both close to the crack tip and away from the crack, with potentially differing implications for the burrower. Microcracks near the major crack can expand the fracture process zone (FPZ), which may be important for long-term burrow creation. With more microcracking, burrows are less likely to close after the burrower passes. Microcracking that occurs away from the main crack is unlikely to benefit the burrower, unless the microcrack joins with the major crack (e.g. electronic supplementary material, appendix S6, figures S14c and S15d).. To more closely examine the impact of loading on microcrack generation as it relates to bioturbation, we categorize microcracks into the following two types: (i) those in the FPZ and (ii) those away from the major crack. Figure 6c,d shows average normalized microcrack length, respectively, in and away from FPZ, for toughness CoVs ranging between 0.1 and 0.4 and subjected to focused and symmetric loading. Within the FPZ, there were significantly fewer microcracks under focused loading compared to that of the symmetric loading across all CoV values (figure 6c; *t*‐test, all p<0.05). Surprisingly, the CoV of fracture toughness did not affect the microcrack length in the FPZ under symmetric loading (linear regression, p=0.645) and resulted in a slight increase in microcracks with CoV under focused loading (linear regression, p=0.087). Focused loading also results in a reduction of microcracks away from the FPZ (*t*‐test, all p<0.05). For both loading scenarios, microcracking away from the crack tip increases significantly with CoV (linear regression, p<0.05).

### 3.4. Iterative crack propagation shows crack steering through asymmetrical force application

**Figure 7.**
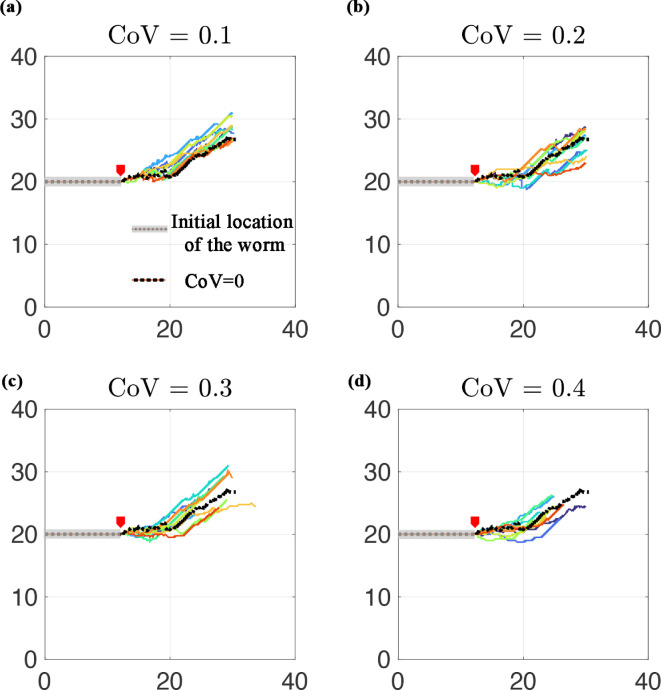
Crack trajectory in 10 realizations of random heterogeneous sediments subjected to asymmetric head pressure shown in figure 1j for sediments with (a) CoV = 0.1, (b) CoV = 0.2, (c) CoV = 0.3 and (d) CoV = 0.4, compared to the reference homogeneous media with CoV = 0 (black dotted line). The top-to-bottom loading ratio is set at 1.5/0.5. All sediments have KIc=200 Pa$/\sqrt{\text{m}}$. The grey area represents the initial notch (the space occupied by the worm at the onset of the simulation), and the red arrow labels the starting point of the crack tip.

Finally, we re-examine the potential for crack steering via crack propagation simulation, specifically whether the interaction between asymmetric pressure and heterogeneity of fracture toughness could influence the direction of crack propagation (hypothesis H3b). To this end, the head region of the two sides of the burrow walls is subjected to asymmetric pressure to simulate a turning behaviour, as illustrated in figure 1j. In contrast to our previous study, which showed a very small crack path deviation (1 degree) under similar asymmetric loading [10], here we find that the crack deviates by 25.5 ± 4.0 degrees (mean ± s.d.) over 18 mm of crack growth at a CoV of 0.2 (figure 7).

We initially predicted that heterogeneity in the fracture toughness field might contribute to crack steering, which was surprisingly small in our previous models in a homogeneous material [10]. However, for all CoV values, the burrow trajectories overlap with the trajectory in the homogeneous material (CoV = 0; depicted by the black line in all panels of figure 7) when subjected to the same ratio of asymmetric loading (figure 7). Comparison of turning angle across the four values of CoV showed significant differences among the means (one-way ANOVA, *p* = 0.02), but does not explain very much of the variability ($R^{2}=0.013$). Although the difference is small, the turning angle was significantly higher at CoV = 0.1 than at 0.4 (pairwise Tukey test, α = 0.05), in contrast to our hypothesis that variability would enable crack turning.

It should be noted that, unlike our previous study [10], which only accounted for the initial crack propagation direction, the LEM enables simulation of incremental crack propagation. More specifically, once the asymmetric pressure creates a crack surface in the material, the fracture-induced heterogeneity is also captured in our simulation, in contrast to the previous modelling approach. That is why the crack pattern is tortuous even for homogeneous sediments.

### 3.5. Observations of microcracking and hydraulic fracture in natural sediments

**Figure 8.**
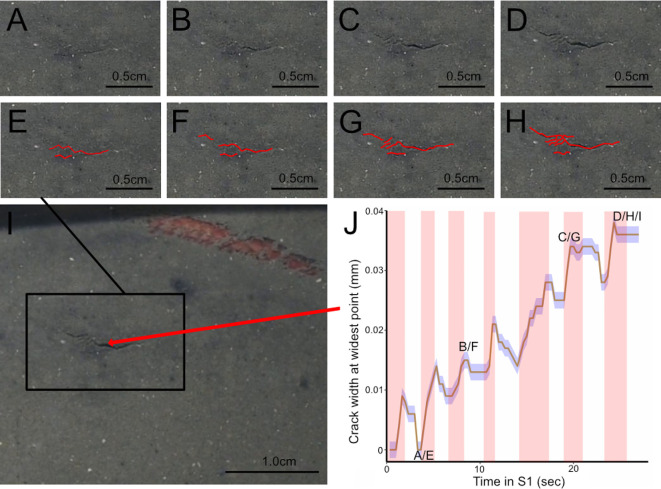
Crack propagation occurs centimetres away from burrowing *A. virens*, synchronously with the eversion and retraction of its pharynx (electronic supplementary material, appendix S7, video S1). Images (A–D): crack formation and propagation are visible within a region of interest (black box in (I)) away from the worm (electronic supplementary material, video S1). Images (A–D) were captured at 3, 9, 20 and 26 s of the 27 s duration of electronic supplementary material, appendix S7, video S1. Images (E–H) correspond to (A–D), with all cracks within the region of interest traced in red. (I) Zoomed out view of (C) showing the burrowing worm and a black outline around the region of interest in images (A–H). (J) Maximum crack width (solid line) at the widest point; see image (I) occurs with the eversion (red shaded regions) and retraction (unshaded regions) of the pharynx, respectively. Blue shading indicates the s.d. of three measurements of crack width. Measurements were taken from three randomly selected images, and s.d. was averaged and applied to the duration of the video, as s.d. did not differ greatly between images. Scale bars are shown at the bottom right of each panel.

In most of the videos recorded of worms burrowing in laminar ant farm tanks, cracks can be seen opening up that do not appear to be connected to a main burrow crack, consistent with microcracking. However, in the majority of these instances, the worm is not visible, making it difficult to confirm that the newly formed crack is unconnected to the main burrow crack. As such, we focused our analysis on video segments in which the worm was entering the sediment, such that the entire position of the worm and thus the main burrow crack was known. Of these seven examples, three clearly show cracks opening that are separate from the main burrow crack (by 5−20 mm). These propagation events, in which cracks widen, propagate and sometimes branch, are concurrent with the repetitive eversion of the worm’s pharynx (electronic supplementary material, appendix S7, video S1; figure 8). We interpret this evidence of crack propagation away from the main burrow crack synchronous with pharynx eversions and retractions (figure 8A–J) to be consistent with the clustered microcracking occurring far from the FPZ in modelling results (figure 2).

Obtaining video footage of ‘hydraulic fracture’, in which crack propagation extended well in front of the everted pharynx, was comparatively difficult given our experimental set-up. As worms mostly preferred to burrow in the very middle of the tank, most pharynx eversions were detected via the bulging of the silicone—such that we could not see the pharynx itself and did not know its exact position. Despite this limited visibility, we were still able to isolate a clip of a burrowing worm that shows the position of the fully everted pharynx as a silicone bulge with movement of surrounding sediment away from the bulge and associated crack branching and extension far in front of the worm (electronic supplementary material, appendix S7, video S2). Furthermore, we observed water flowing through the branched crack, supporting the argument that, in this case, the mechanism behind crack extension is hydraulic fracture (electronic supplementary material, appendix S7,video S2).

## 4. Discussion

### 4.1. Burrow extension by fracture causes microcracking in low-toughness regions of the surrounding sediment

Although microcracking has been observed around the crack tip of worms burrowing in natural sediments [10], this is the first study that has successfully modelled microcracking around simulated burrowing worms. The prevalence of microcracking, especially at substantial distances away from the crack tip, was surprizing and led us to carefully examine natural sediments throughout the entire laminar ant farm tanks rather than focusing only in the vicinity of the crack tip. Both model results and observations of burrowing worms show microcracks forming and propagating at distances ranging from centimetres to tens of centimetres away from the main burrow crack. In model results, microcracking occurs in regions of low fracture toughness and can occur in the farthest corners of the modelling domain from the crack tip (>30 cm away; figure 6). Observations of microcracking in the sediment around burrowing worms in our 2D ant farm tanks are consistent with simulation results, occurring approximately 5−10 cm from the worm in regions that appear less compact (figure 8). These microcracks propagate iteratively in conjunction with pharynx eversions and main burrow crack extension (figure 8; electronic supplementary material, appendix S7, video S1).

Both the numerical model and the ant farm tanks differ from *in situ* sediments in several potentially important ways. First, the 2D geometry sandwiched between layers of gelatin likely results in increased pressure from pharynx eversion that propagates further than in natural three-dimensional sediments, potentially opening up microcracks at greater distances from the worm than would occur in natural sediments. Assumptions in the model may also lead to microcracking at greater distances than in natural sediments. The model assumes linear elasticity, whereas natural sediments exhibit hysteresis: stored elastic energy is likely dissipated through grain–grain interactions [2], potentially reducing the distance from the FPZ that the energy available for fracture is sufficient for microcracking to occur. The model also ignores effective stress and depth dependence of fracture toughness in natural sediments; fracture toughness increases with depth in the surface layer (approx. 20 cm) of sediment, although this increase is nonlinear, in some cases increasing sharply at depths potentially corresponding with deposition layers or bioturbation [32]. Thus, microcracking may decrease with depth in sediments, in contrast to the relatively uniform distribution predicted by our model (see electronic supplementary material, appendix S5). The amount and spatial distribution of variability in fracture toughness likely also differ between our experimental tanks and natural sediments. In the experiment, the sediment was homogenized and poured into the tanks to settle, resulting in less heterogeneity than most natural sediments. However, this layering and dewatering can sometimes result in horizontal cracks or weak zones in the sediment, as sediments can be constrained by the tank walls and not settle evenly. Similar weak zones or regions of low fracture toughness likely occur in natural sediments, both through storm disturbance and layered deposition and through burrows that partially close or collapse. Our modelling results indicate that microcracking far from the FPZ increases with increasing variability in fracture toughness (figure 6d); microcracking may be reduced by homogenization in experimental tanks and be more localized around horizontal weak zones resulting from dewatering compared to natural sediments.

### 4.2. Variability in fracture toughness affects microcracking and crack branching in different ways

We initially hypothesized that microcracking and crack branching would show similar patterns, both increasing with variability in fracture toughness (H1) and decreasing with mean fracture toughness, as higher fracture toughness requires more energy for cracks to grow (H2). We found more support for our hypothesis that microcracking and crack branching depend on variability in fracture toughness than on the mean value (figure 3). However, in both cases, the model results showed greater complexity than we anticipated. It is important to note that while our modelling approach effectively incorporates heterogeneity in material properties and is therefore well-suited for testing H1, it is less equipped to test H2. Specifically, we expected that materials with low fracture toughness would experience more crack branching and microcracking, as more energy is available for fracture under the same loading conditions. However, the LEM simulations are performed under quasi-static conditions, which do not account for the effect of speed of loading on dynamic fracture behaviour. In such quasi-static conditions, crack initiation and propagation are governed by the ratio of applied stress to fracture toughness. As a result, mean fracture toughness determines the load required to initiate or propagate a crack but does not affect the crack pattern, including the formation of microcracks and branches. In other words, since the model does not incorporate time, it cannot simulate the build-up and sudden release of stored energy that could occur if the force applied by the worm exceeds the rate at which energy is dissipated through crack propagation. Although worms move slowly enough that dynamic fracture effects may be negligible, if time-dependent processes are significant, the model may underestimate the influence of fracture toughness on crack branching and microcracking.

Our expectation that microcracking and crack branching would show similar patterns was likely influenced by our implicit assumption that microcracking would be confined to the FPZ, close to the crack tip, as observed in previous studies of microcracking around burrowers [10]. Microcracks, however, were spread throughout the simulation domain (figure 2). The differences in the scales of these two processes potentially explain their distinct relationships with variability in fracture toughness. Crack branching appears to be a localized process that depends on the local gradient in fracture toughness, whereas microcracking showed different relationships with variability in fracture toughness inside versus outside the FPZ. Microcracking also depends on the stress application, with less microcracking observed when stress is concentrated at the crack tip (figure 6). There was considerable variability in microcracking across the different simulated fracture toughness fields. Increasing the number of random realizations would likely clarify the patterns, but variability is also inherent in heterogeneous material. The assumptions of our simplified model, e.g. 2D, with no hysteresis or depth dependence, likely affect the distribution of microcracks, adding uncertainty to potential attempts to quantify these patterns.

### 4.3. Implications for burrowing strategies in natural, heterogeneous sediments

**Figure 9.**
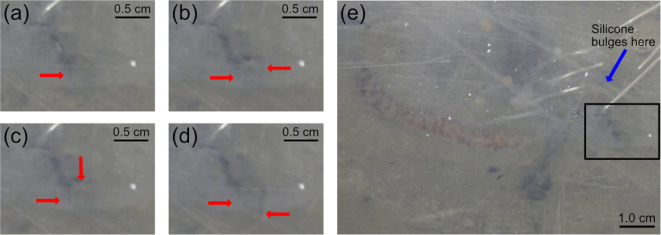
Pharynx eversion and subsequent crack branching and extension out in front of the worm, likely driven by hydraulic fracture and following a path of lowest fracture toughness (electronic supplementary material,appendix S7, video S2). (a) Silicone bulge and concurrent sediment movement indicate a pharynx eversion (8 s mark of electronic supplementary material, appendix S7, video S2). (b) The crack then branches to the right (0.4 s after image (a)), (c) continues extending both rightwards and downwards (0.5 s after (a)) and then continues to extend downwards in (d) (0.8 s after (a)). Red arrows point to crack tips. (e) The region of images (a–d) in the context of the burrowing worm (blue arrow shows location of pharynx). Scale bars are shown at the bottom right of each panel.

Interestingly, both the model results and observations suggest that hydraulic fracture may play an important role in burrow extension in heterogeneous sediments. Many diverse burrowing animals have been described as using body expansions to anchor part of the body while another part moves forward [6], and it has been suggested that these anchors may also act as seals to drive water forward to facilitate burrowing [5]. As many burrowers actively irrigate their burrows, it would not be surprizing if pumping oxygenated water into the burrow served a secondary function in burrowing. Water movement has not been observed around animals burrowing in transparent, homogeneous gelatin [2], although in the laminar ant farm tanks, water flow is apparent from the movement of suspended particles of sediment (electronic supplementary material, appendix S7, video S2), which are absent in gelatin. Here we show evidence of hydraulic fracture in natural sediments driving crack propagation along a tortuous path, presumably the path of least resistance, extending well ahead of the worm (Figure 9). Our modelling results show that focusing stress at the crack tip reduces microcracking away from the burrow (figure 6), providing an additional rationale for the use of hydraulic fracture. The release of energy through microcracking may increase the energetic cost of burrowing, although burrowers move slowly enough that the energetic cost of burrowing is not high, so this may not be an important driver of behaviour [4]. Microcracking in front of the crack tip has been observed around worms burrowing horizontally in natural sediments [10]. These microcracks can fuse with the main burrow crack, observed in simulation results as sudden drops in the microcrack length (figure 3 inset and figure 6). This microcrack fusion may steer the burrow towards regions of low toughness more effectively than if the main crack simply iteratively followed the path of least resistance.

That burrow extension by fracture can open cracks away from the burrower suggests a mechanism for interactions among burrowers in this opaque medium. Pressure signals from burrower activities such as irrigation have been measured 20 cm away from the burrower [33]. Whether these pressure signals are detected by other worms, e.g. between predators and prey, has not been explored. Our results suggest that weak regions, e.g. relic burrows, may be opened to release stored potential energy, raising a possible second mechanosensory cue that could drive interactions within the benthos. Microcracking is driven by increased stress in the material; either the increased stress or the formation of microcracks may be a signal to predators that a burrower is nearby. Predator–prey interactions in sediments are poorly understood, and how predators detect prey is unknown. If these signals are mechanical, then hydraulic fracture may offer a more stealthy way of burrowing for potential prey. We have previously suggested that the mechanism of crack propagation raises the possibility of predators finding prey more easily, as a crack would follow the path of least resistance, e.g. along a burrow made by a potential prey organism [5]. These results expand this possibility, suggesting that relic burrows may be maintained through the activities of burrowers, even some distance away.

### 4.4. Insight into mechanisms of ecosystem engineering

Based on previous modelling results showing that variability in fracture toughness is important in increasing crack path tortuosity, we suggested that feedback between burrowers increasing sediment heterogeneity and the implications of heterogeneity on burrowers provide a potential mechanism for ecosystem engineering [10]. These results provide further support by showing that crack branching and microcracking also depend on the CoV in fracture toughness. That microcracking appears to be more prevalent than we expected away from the crack tip region is further evidence for burrowing animals increasing sediment heterogeneity. There are several potentially important implications of these feedback mechanisms. First, animal–sediment interactions may differ across different sediment conditions. In relatively homogeneous muds with few burrowers, e.g. after deposition or physical disturbance, low variability in fracture toughness would suggest low occurrence of crack branching and microcracking, especially away from the main burrow. Cracks would be expected to propagate relatively straight, with navigation driven by asymmetric stress application to steer the burrows rather than by crack branching. As a result, mixing of sediments would occur relatively slowly, and sediments would remain relatively homogeneous until sufficient burrowing occurred to increase heterogeneity. In contrast, in more heterogeneous muds with well-established communities of burrowers, higher variability in fracture toughness would result in more crack branching, more crack opening away from the main burrow and, therefore, more sediment mixing and a more rapid increase in heterogeneity. This suggests that bioturbation may not increase linearly with infaunal abundance, but rather exhibit a transition from low bioturbation in homogeneous sediments with low abundance to high bioturbation with greater dependence on infaunal abundance in more heterogeneous, bioturbated sediments. This transition may be exacerbated by burrower behaviour—if navigation is easier in heterogeneous sediments, burrowers may be more active, further contributing to increased heterogeneity. At the farthest extreme of high variability in fracture toughness, e.g. in very highly bioturbated sediments, stress applied to burrow walls may primarily be released through opening of weak regions away from the main crack (figure 6b under symmetric stress), making extension of the main burrow by fracture less feasible. This could explain why the surface layer of well-bioturbated sediments exhibits mechanical behaviour more akin to soft aggregates than to a cohesive elastic solid. Second, crack opening and extension in sediments away from the main burrow, such as re-opening of previous burrows, increase the heterogeneity of not only physical properties but also, consequently, the chemical properties of sediments. If nearby relic burrows are opened through the burrowing activities of nearby worms, these pressure fluctuations could drive water flow and flush relic burrows, altering geochemical gradients and processes [34].

One limitation of our modelling approach in understanding bioturbation is that we started with a prescribed variability in fracture toughness without any *a priori* knowledge of the actual magnitudes and patterns of variability in fracture toughness in natural sediments. This variability is challenging to measure on small spatial scales. There are very few measurements of fracture toughness in natural sediments [35–37], and current methods to measure fracture toughness have a spatial resolution of approximately 5 mm [37]; thus, little is known about the small-scale variability in fracture toughness in natural sediments. Variability is presumably driven by the interactions between burrowing animals opening cracks in the cohesive matrix and microbially mediated recovery of cohesion [38]. Our modelling approach captures burrower-created heterogeneity, as demonstrated by the stochastic turning of crack paths in homogeneous material (Figure 7), and has potential for further exploring processes that lead to bioturbation. We intentionally restricted our simulations to one burrow extended through a material with prescribed properties to examine the effect of those properties on the crack path. However, the hybrid energy-based LEM provides the means to model the process of bioturbation, although it is computationally expensive. This can be achieved by simulating multiple crack paths through an initially relatively homogeneous material to examine the interaction of burrows and by modelling the cohesion recovery through the healing of cracks to explore the complementary question of how burrowers alter material heterogeneity, particularly in terms of fracture toughness variability.

Moreover, in this study, we examined the impact of the mean and CoV of fracture toughness and briefly considered CL. Our results suggest that CL—a property of the two-point correlation function that indicates how points within a stochastic domain are correlated—can play an important role, particularly in crack branching. However, we did not have sufficient simulation results with varying CLs to fully investigate this aspect. Recent work [26] has demonstrated the broader influence of the two-point correlation function, extending beyond CL. The observed influence of CL in our study aligns with these findings, highlighting the potential importance of CL, or correlation structure in general, on bioturbation. Further investigation into how the autocorrelation function’s structure (with CL as its simplest and most fundamental property) impacts bioturbation processes, such as microcracking, crack branching and steering angle, would be valuable. Additionally, our LEM simulations use harmonic potentials and rely on the Griffith criteria and LEFM [25]. Incorporating plastic deformation before fracture, by utilizing non-harmonic potentials within the PMF-based LEM framework, would provide a more comprehensive understanding of fracture processes in non-brittle heterogeneous sediments. Finally, accounting for rate-dependent fracture energy to capture dynamic fracture processes would be useful for examining how microcracking and branching are influenced by burrowing speed and variability in mean fracture toughness.

Integration of improved methods to measure fracture toughness [10,30] with modelling of fracture in heterogeneous materials is crucial for the development of a mechanistic understanding of bioturbation in muddy sediments, particularly the interactive relationship between burrowing activity and sediment properties—how burrowers modify sediment heterogeneity and how these changes influence the mechanical properties of sediments and further bioturbation.

## 5. Conclusions

Crack branching events allow burrowers in muddy sediments to change directions and navigate more easily. Our modelling results show that, contrary to our hypothesis that more crack branching would occur when fracture toughness was low and highly variable, the crack branching probability was actually highest at intermediate values of both mean fracture toughness and variability in fracture toughness. Both model results and observations of burrowers show microcracks forming and growing at a distance from the main burrow; this microcracking increases sediment heterogeneity and may also contribute to bioturbation and act as a mechanical signal of prey presence to potential predators. Microcracking occurs more in materials with higher fracture toughness, presumably because resistance to fracture of the main burrow allows build-up of stress in the material. Burrowers can reduce this microcracking by focusing stress at the crack tip through hydraulic fracture. These results highlight the need for future research on the magnitude and spatial variability of fracture toughness in natural sediments.

## Data Availability

The data supporting the results of this study are provided in the electronic supplementary material: a PDF supplementary file and the supplementary videos (numerical simulation and actual observation). Electronic supplementary material is available online [39].
